# Enhanced stability and clinical absorption of a form of encapsulated vitamin A for food fortification

**DOI:** 10.1073/pnas.2211534119

**Published:** 2022-12-12

**Authors:** Wen Tang, Jia Zhuang, Aaron C. Anselmo, Xian Xu, Aranda Duan, Ruojie Zhang, James L. Sugarman, Yingying Zeng, Evan Rosenberg, Tyler Graf, Kevin J. McHugh, Stephany Y. Tzeng, Adam M. Behrens, Lisa E. Freed, Lihong Jing, Surangi Jayawardena, Shelley B. Weinstock, Xiao Le, Christopher Sears, James Oxley, John L. Daristotle, Joe Collins, Robert Langer, Ana Jaklenec

**Affiliations:** ^a^David H. Koch Institute for Integrative Cancer Research. Massachusetts Institute of Technology, Cambridge, MA, 02139; ^b^South China Advanced Institute for Soft Matter Science and Technology, School of Emergent Soft Matter, South China University of Technology, Guangzhou 510640, China; ^c^VitaKey Incorporation, Durham, NC 27701; ^d^Rice University, Houston, TX 77005; ^e^Department of Biomedical Engineering, Johns Hopkins University, Baltimore, MD 21231; ^f^Key Laboratory of Colloid, Interface and Chemical Thermodynamics, Institute of Chemistry, Chinese Academy of Sciences, Zhong Guan Cun, Beijing 100190, China; ^g^Weinstock Nutrition, LLC, South Orange, NJ 07079; ^h^Independent Scholar, Belmont, MA 02478; ^i^Southwest Research Institute, San Antonio, TX, 78238

**Keywords:** micronutrient, food fortification, microparticle, encapsulation

## Abstract

Food fortification is an effective strategy to address vitamin A (VitA) deficiency, which is the leading cause of childhood blindness and drastically increases mortality from severe infections. However, VitA food fortification remains challenging due to significant degradation during storage and cooking. We utilized an FDA-approved, thermostable, and pH-responsive basic methacrylate copolymer (BMC) to encapsulate and stabilize VitA in microparticles (MPs). Encapsulation of VitA in VitA-BMC MPs greatly improved stability during simulated cooking conditions and long-term storage. VitA absorption was nine times greater from cooked MPs than from cooked free VitA in rats. In a randomized controlled cross-over study in healthy premenopausal women, VitA was readily released from MPs after consumption and had a similar absorption profile to free VitA. This VitA encapsulation technology will enable global food fortification strategies toward eliminating VitA deficiency.

## Significance

Fortifying common daily foods is an ideal solution to decrease the prevalence of vitamin A (VitA) deficiency. However, VitA food fortification is challenging due to its poor stability under heat,light, moisture, and oxidative conditions and its poor miscibility with water or centrally processed foods, such as flour. It is crucial to address both stability and bioavailability to ensure the efficacy of VitA food fortification. Here, we developed a VitA encapsulation technology that is produced by a commercial process, provides effective protection during storage and cooking, and is bioavailable in humans.

Vitamin A (VitA) plays an essential role in visual health, immune function, and fetal growth and development (1). VitA deficiency (VAD) arises from diets with insufficient fat-soluble micronutrients (MNs) and is currently estimated as the second most common cause of malnutrition, after iron, globally (2). VAD can lead to xerophthalmia (preventable childhood blindness) and weakened host resistance to infection, which can increase the risk of mortality from infectious diseases, such as measles and COVID-19 (3, 4). The WHO estimated that VAD affected 190 million preschool-age children (33.3% of the preschool-age population) and >19 million pregnant women (15.3% of the pregnant population) globally in the period spanning 1995–2005 (5). The most severely affected regions still reached VAD prevalence levels of 48% in sub-Saharan Africa and 44% in South Asia in children in 2013 (6). To reduce the high burden of VAD, a VitA supplementation program was implemented worldwide in 1990 that distributed high-dose VitA capsules every 4–6 mo to over 80% of the total child population in low-income countries (7). This project effectively reduced all-cause mortality caused by severe VAD by 12% (8). However, progress toward VAD elimination was limited to a rate of improvement of only ~0.3% per year from 1990 to 2007, showing that more impactful strategies are required (9, 10).

To raise and maintain serum retinol levels, frequent intake of VitA at physiological doses is proven to be more effective than one or two high doses administered over 6 mo (11). However, VitA food fortification is challenging due to its poor stability, which can lead to poor bioavailability after degradation, and fat solubility, which limits the inclusion of VitA in water-based and dry food matrices (12). To prevent VitA degradation and improve miscibility, VitA has previously been encapsulated within matrices composed of polysaccharides (13), proteins (14), and/or lipids (15); however, these materials provide limited protection during storage and cooking (16–18) and can take up to 3 h to release in the stomach (19). Poor protection and slow release of VitA prevent effective absorption. Therefore, the ideal microparticle (MP) platform for VitA fortification should meet these criteria: i) protect VitA against degradation during storage and cooking; ii) rapidly release VitA in the gastrointestinal tract with high absorption; and iii) readily mix with various food matrices at a tunable concentration to meet the dynamic needs of the target population.

We hypothesized that by encapsulating VitA with a pH-responsive hydrophobic polymer, we could enhance stability during storage and cooking and ensure its rapid release in the gastrointestinal tract for subsequent absorption. A commercially available, FDA-approved basic methacrylate copolymer (BMC), also known as either Eudragit® E PO or GRAS-status Eudraguard®, was identified from our previous work (20). BMC is generally regarded as safe with an acceptable daily intake of 20 mg/kg body weight (21). VitA-encapsulated BMC MPs were prepared by emulsion at the laboratory scale and by a commercial process at the kilogram scale. Our VitA-BMC-S MPs readily mix with flour and bouillon cubes and demonstrate enhanced stability under cooking and long-term storage conditions (over 12 mo) in comparison to a leading commercial encapsulated VitA product. The bioavailability of VitA from VitA-BMC MPs was first demonstrated in a rodent model, resulting in a ninefold increase in the accumulation of VitA in the liver from cooked VitA-BMC MPs, as compared to cooked unencapsulated free VitA. In a human clinical study, the absorption of VitA from bread fortified with VitA-BMC-S MPs was investigated, with or without the codelivery of encapsulated iron sulfate (FeSO_4_) and folic acid (FA), MNs that children and pregnant women globally are also often deficient in (22, 23). The results indicate that VitA is readily released and absorbed from VitA-BMC-S MPs, and the codelivery of encapsulated iron and free FA does not influence the absorption of VitA. In total, we demonstrated scalable production of MP-encapsulated VitA with enhanced stability and good bioavailability in humans, which could potentially mitigate the high burden of VAD and be codelivered with other MNs.

## Results

### Formulation of MPs and Pilot Stability Study.

**Fig. 1.**
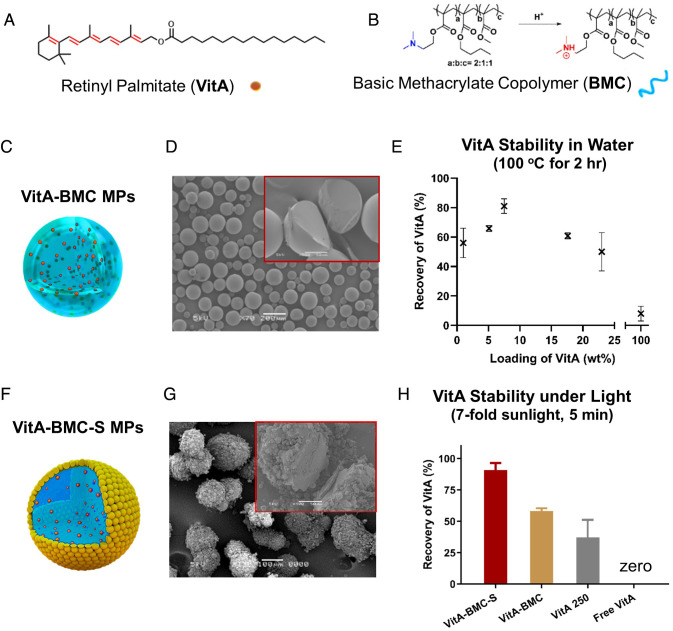
VitA-BMC and VitA-BMC-S MPs were prepared at laboratory and commercial scale, respectively. The VitA stability was studied in boiling water and under light irradiation. Chemical structures of (*A*) retinyl palmitate (VitA) and (*B*) BMC. VitA-BMC MPs prepared by emulsion method at laboratory scale are shown (*C*) in a schematic, (*D*) as SEM images (Scale bar represents 200 μm), and the cross-section of a VitA-BMC MP (inset, Scale bar represents 50 μm). (*E*) The stability of VitA measured as percent recovery in boiling water for 2 h is enhanced by encapsulation compared to free VitA (i.e., 100% loading). The loading percentage of VitA in the MPs affects the stability, and the 7.5 wt% VitA in VitA-BMC MPs provides the highest recovery. VitA-BMC-S MPs prepared by spinning disk at premanufacture scale (*F*) shown as a schematic, (*G*) SEM images of the MPs (Scale bar represents 100 μm), and the cross-section of a VitA-BMC-S MPs (inset, Scale bar represents 50 μm). (*H*) Stability of VitA after irradiation under simulated sunlight beam (sevenfold intensity, 5 mins) on a sample stage at 10°C. All free VitA is degraded and the highest recovery rate of VitA-BMC-S MPs is 91 ± 5% VitA.

VitA was initially encapsulated (Fig. 1*A*) in BMC (Fig. 1*B*) using an oil-in-water emulsion process in the lab (*SI Appendix*, Fig. S1*A*). The resulting VitA-BMC MPs (Fig. 1*C*) exhibited a homogenous internal structure (Fig. 1*D*) and a size distribution of 168 ± 47 μm (*SI Appendix*, Fig. S1*B*), which is desirable for the food fortification of flour and bouillon cubes. The loading amount of VitA was tuned from 1 to 23 wt% by changing the feed ratio of VitA to BMC, which is important for fortification dosing (*SI Appendix*, Table S1). The stability of VitA in boiling water for 2 h was tested. Although the recovery of free VitA was only 9 ± 5%, the recovery of all 1–23 wt% loaded VitA-BMC MPs exceeded 50%, highlighting the effective protection of VitA (Fig. 1*E*). The 7.5 wt% VitA-BMC MP had the highest recovery of 81 ± 5%, a ninefold enhancement over free VitA. This optimal loading that provides the highest VitA recovery after boiling could be the result of the antioxidant and plasticizer effects of VitA. When the loading of VitA in BMC increased, the radical scavenging activity increased, while the glass transition temperature (Tg) of VitA-BMC mixture decreased (*SI Appendix*, Fig. S2). The lower Tg indicates higher flowability of polymer segments, which may result in higher VitA degradation.

In order to produce the particles at scale, a pilot process was developed using a spinning-disk atomizer (*SI Appendix*, Fig. S3*A*). We first applied the optimal mass feeding ratio of 1:10 (VitA:BMC) in the spinning-disk process. However, the afforded VitA-BMC MPs were tacky and tended to aggregate. We suspect the agglomeration of MPs is due to the plasticization of BMC by VitA (*SI Appendix*, Fig. S2). Therefore, we collected the MPs on a starch (S) bed to coat the particle surface with starch and prevent MPs from agglomerating. After sieving off extra starch and agglomerates, the VitA-BMC-S MPs were obtained, whose idealized illustration is shown in Fig. 1*F*. The VitA-BMC-S MPs were 141 ± 50 μm in diameter (*SI Appendix*, Fig. S3*B*) characterized by the Coulter counter. The inside of the VitA-BMC MPs was similar to VitA-BMC MPs according to the cross-sectional SEM image (inset of Fig. 1*G*), and the starch particles (~10 μm) were randomly covered on the outer layer of the MPs (Fig. 1*G* and *SI Appendix*, Fig. S3 *C*–*F*). Surprisingly, the VitA-BMC-S MPs exhibited improved protection against sunlight irradiation with a recovery of 91 ± 5% of VitA compared to recovery from VitA-BMC MPs at 58 ± 2%. All formulations were compared to a commercially available encapsulated VitA (VitA 250 MPs) that contains the antioxidant butylated hydroxytoluene (BHT), which is currently the VitA fortificant standard (24). VitA 250 MPs showed a recovery of 37 ± 14% VitA under the same irradiation, while free VitA was completely degraded (Fig. 1*H*). We then removed organic solvents from the process to make it more environmentally friendly and reduce production costs. An aqueous process was developed using a spray dryer, which was fed with an aqueous emulsion containing VitA, BMC, and other additives. Because no organic solvent was involved during this process, the resulting particles were referred to as VitA-BMC (aqueous) MPs. The formulations of MPs used in this paper are summarized in *SI Appendix*, Table S2.

### pH-Controlled Release.

**Fig. 2.**
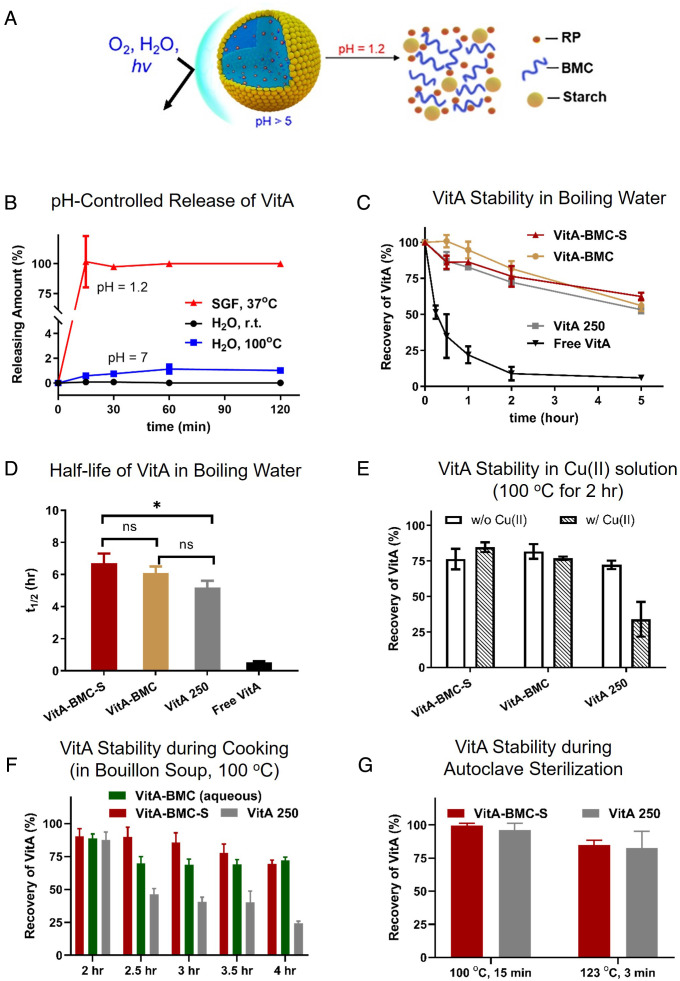
VitA-BMC-S MPs exhibit fast release in the stomach (pH = 1.2) and protect VitA from degradation by heat, moisture, and oxidative species during cooking. (*A*) Schematic representation illustrates VitA protection from the surrounding environment and the pH-responsive release of VitA from VitA-BMC-S MPs in SGF. (*B*) No VitA released from VitA-BMC-S MPs in deionized water at room temperature (black curve) or 100°C (blue curve) up to 2 h. All the VitA released within 15 mins in 37°C SGF, pH 1.2 (red curve). (*C*) VitA recovery of VitA-BMC-S and VitA 250 MPs in 100°C water up to 5 h. VitA-BMC-S and VitA-BMC MPs stabilize VitA equally well, and VitA-BMC-S is significantly different from the commercial product VitA 250 (*P < 0.05) in water as indicated by half-life (*t*_1/2_) values (*D*). (*E*) VitA stability in boiling water with or without 65 mM Cu(II). The existence of Cu(II) does not change the stability of VitA in VitA-BMC and VitA-BMC-S MPs, while the commercial VitA 250 MPs degrades due to the Cu-induced oxidation. (*F*) VitA stability of MPs in bouillon soup at 100°C for 2–4 h. VitA-BMC-S MPs and VitA-BMC (aqueous) MPs provide better stability than VitA 250 MPs when the cooking time is longer than 2 h. (*G*) VitA stability of MPs in water under autoclave conditions, 100°C for 15 mins and 123°C for 3 mins. VitA-BMC-S MPs provide compatible stability in comparison with VitA 250 MPs.

In vitro release studies confirmed that immediately after being submerged in simulated gastric fluid (SGF, pH = 1.2), both the VitA-BMC (*SI Appendix*, Fig. S4) and VitA-BMC-S MPs quickly (<15 min) dissolve and release VitA (Fig. 2*B*). The release of VitA was not detected in water at neutral pH at room temperature or at 100°C for over 2 h (Fig. 2*B*, black and blue curves). This pH-responsive burst release ensures strong protection during storage and cooking and rapid release in the stomach.

### VitA Stability During Cooking.

Degradation leads to less available VitA for absorption after ingesting VitA-fortified food. Hence, we investigated the thermal stability of VitA-BMC and VitA-BMC-S MPs, in boiling water as a simulated cooking condition, and compared them with free VitA and the VitA 250 MPs, respectively. The VitA recovery from VitA-BMC and VitA-BMC-S MPs in boiling water after 5 h is 56 ± 4% and 62 ± 3%, respectively, while about 50% of free VitA quickly degrades after 15 min in boiling water and only 6 ± 1% remains after 5 h (Fig. 2*C*). The half-life (*t_1/2_*) of free VitA (*t_1/2_* = 0.52 ± 0.07 h) is 11-fold shorter than that of VitA-BMC-S MPs (*t_1/2_* = 6.7 ± 0.6 h) and VitA-BMC MPs (*t_1/2_* = 6.1 ± 0.4 h) (Fig. 2*D*). VitA-BMC-S MPs also show slightly better protection (i.e., longer half-life) than the VitA 250 MPs (*t_1/2_* = 5.2 ± 0.4 h). It is worth noting that VitA-BMC and VitA-BMC-S MPs do not contain additional antioxidants, while VitA 250 MPs are stabilized with BHT.

Oxidative species, such as Cu(II), that are naturally present in foods can induce VitA degradation (25). Therefore, the stability of VitA in boiling water with Cu(II) for 2 h was studied. Cu(II) did not change the VitA recovery from VitA-BMC and VitA-BMC-S MPs, whereas the VitA recovered from VitA 250 MPs sharply decreased (Fig. 2*E*). Bouillon cubes are widely consumed in Africa and were identified as a promising vehicle for MN fortification, but commercially available options are limited (26). We tested the stability of VitA in soup made of bouillon cubes at 100°C for 2–4 h (Fig. 2*F*). The VitA-BMC-S and VitA-BMC (aqueous) MPs provided strong protection of VitA with 69 ± 3% and 72 ± 2% recovery, respectively, even after 4 h of cooking, which is about three times that of VitA 250 MPs at 24 ± 2%. The stability of VitA was further challenged under autoclave conditions of extremely high temperature and humidity. After being autoclaved at 100°C for 15 mins and at 123°C for 3 mins, 99 ± 2% and 85 ± 4% VitA were recovered from VitA-BMC-S MPs, respectively, which is comparable to that of VitA 250. Collectively, the VitA-BMC-S MPs protect VitA against heat, water, and oxidative species in cooking processes and significantly outperform the currently available commercial product.

### VitA Stability During Storage.

**Fig. 3.**
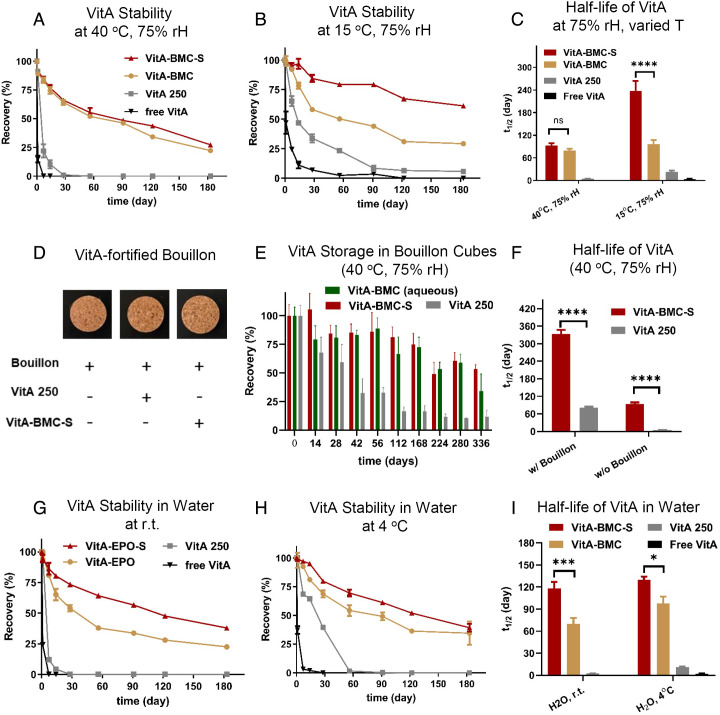
VitA-BMC MPs provide outstanding protection of VitA as dry powders alone or mixed into bouillon cubes when stored long term at high humidity, at high temperature, or as an aqueous suspension. Percent recovery of VitA was measured from VitA-encapsulated MPs stored as open-air dry powders over the course of 6 mo at (*A*) 40°C, 75% rH and (*B*) 15°C, 75% rH. Free VitA is the negative control, and VitA 250 MPs is the current commercial product control. Fig. (*A*) and (*B*) share the same figure legends. (*C*) Calculated half-life time (*t*_1/2_) of VitA in MPs as dry powders at different conditions, ns *P* > 0.05 and ****P < 0.0001. (*D*) VitA-BMC-S and the commercial VitA 250 MPs were mixed into bouillon cubes and did not change the appearance of the samples. (*E*) Percent recovery of VitA from VitA-BMC-S, VitA-BMC (aqueous), and the commercial VitA 250 MPs within bouillon cubes at 40°C, 75% rH. VitA is better preserved in VitA-BMC-S and VitA-BMC-S (aqueous) MPs than that in VitA 250 MPs. (*F*) Calculated *t*_1/2_ of VitA in MPs within or without bouillon cubes at 40°C, 75% rH, ****P < 0.0001. Mixing within bouillon cubes enhances the stability of VitA for both MPs, and VitA-BMC-S MPs in bouillon cubes elongated the *t*_1/2_to 333 ± 14 d. The percent recovery of VitA from VitA-BMC-S and VitA 250 MPs after 6 mo storage dispersed in water at either (*G*) room temperature (~20–22°C) or (*H*) 4°C. Fig. (*G*) and (*H*) share the same figure legends. (*I*) Calculated *t*_1/2_ of VitA in MPs in water at different conditions, *P < 0.05 and ***P < 0.001. Light was obstructed for all the samples.

Losses of free VitA in fortified foods during distribution and storage are often substantial. The guided condition set of accelerated stability tests of pharmaceutical products concerning hot climatic zones or the global market by the WHO is 75% relative humidity (rH) at 40°C over 6 mo (27). Unlike most previous studies of VitA fortificants in sealed packages, we directly exposed MPs to the environment to eliminate the difference caused by packaging. Free VitA (Fig. 3*A*, black curve) completely degraded within 1 wk at 40°C and 75% rH and commercial VitA 250 MPs degraded within 1 mo (Fig. 3*A*, gray curve). VitA-BMC and VitA-BMC-S MPs provided the best protection (Fig. 3*A*, yellow and red curves, respectively) with a calculated half-life (*t_1/2_*) of 79 ± 5 and 93 ± 6 d, respectively (Fig. 3*C*). After 6 mo at 40°C and 75% rH, the VitA 250 and VitA-BMC MPs formed agglomerates, while the VitA-BMC-S MPs retained their flowability, which is beneficial for subsequent mixing with food. At a lower temperature, 15°C and 75% rH, the stability of VitA-BMC-S MPs is better than that of all the other particle formulations (Fig. 3*B*) with a *t_1/2_* of 238 ± 26 d (Fig. 3*C*).

We also tested the storage stability of VitA in bouillon cubes. The addition of MPs did not change the appearance of bouillon cubes (Fig. 3*D*). VitA-BMC-S and VitA-BMC (aqueous) MPs show stronger stability than VitA 250 MPs during 12 mo of storage at 40°C and 75% rH (Fig. 3*E*). After 12 mo, VitA-BMC-S MPs retained 54 ± 4% VitA, which was 4.5 times that of VitA 250 MPs. The VitA is more stable when mixed within bouillon cubes, and the calculated *t_1/2_* of VitA-BMC-S, VitA-BMC (aqueous), and VitA 250 MPs is 333 ± 14, 285 ± 11, and 81 ± 4 d, respectively (Fig. 3*F*). The *t_1/2_* of VitA-BMC-S MPs is over four times that of VitA 250 MPs, indicating a significant enhancement in VitA stability. To explore the possibility of adding the MPs into a beverage, the MPs were dispersed into water at room temperature (~20–22°C) and 4°C, and the recovery of VitA was quantified (Fig. 3 *G* and *H*, respectively). VitA-BMC and VitA-BMC-S MPs are more stable in water than VitA 250 MPs and free VitA. VitA-BMC-S MPs have the longest *t_1/2_* of 130 ± 9 d in water at 4°C, 12 times that of VitA 250 MPs at 11 ± 1 d (Fig. 3*I*). Overall, the VitA-BMC-S MPs demonstrate the greatest storage stability at higher temperatures, humidity, and moisture content.

### Absorption of VitA from VitA-BMC MPs In Vivo.

**Fig. 4.**
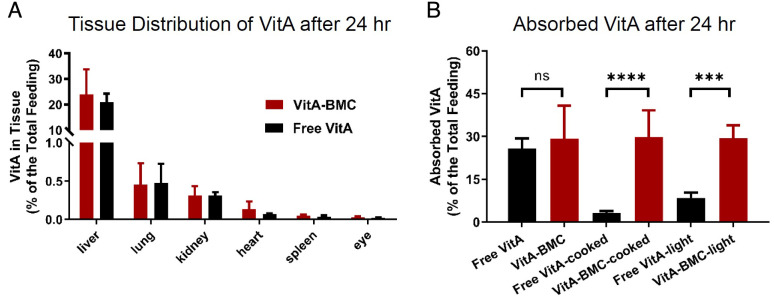
Absorption in rats of tritium-labeled free retinyl palmitate (T-VitA) and T-VitA-BMC MPs after administration by gavage (10 μCi/rat). (*A*) There was no significant difference in tissue distribution of T-VitA absorbed from free T-VitA and T-VitA-BMC MPs (*n* = 6). (*B*) Pretreated (cooked or light irradiated) T-VitA absorption levels (as percentage of the total feeding) in tissues after 24 h. The total absorption is the sum of T-VitA in the small intestine, liver, lung, spleen, kidney, heart, and eye. The cooking (boiling in water for 1 h) and light irradiation (simulated sunlight beam with sevenfold intensity at 10°C for 5 min) processes did not influence the bioavailability of T-VitA-BMC MPs, but dramatically decreased the bioavailability of free T-VitA (ns indicates *P* > 0.05, ***P < 0.001, and ****P < 0.0001).

To confirm the bioavailability of VitA-BMC MPs, we used female Wistar rats to trace the absorbed VitA in rat tissues using tritium-labeled retinyl palmitate (T-VitA). T-VitA-BMC MPs (with or without cooking and photoirradiation treatment) were orally administrated to rats by gavage. The tissue distribution of VitA was quantified after 24 h (Fig. 4*A*). Above 95% of the absorbed VitA was found to accumulate in the liver, the major storage place of VitA, which is consistent with the previous VitA metabolism study (28). In the rats fed with VitA-BMC MPs and free VitA, no statistical difference of VitA in the main organs and the total absorption was found (Fig. 4*A*), indicating that encapsulation in BMC does not influence absorption and bioavailability. Protection by BMC encapsulation was observed in the in vivo results as well. The absorption of VitA dramatically decreased in the rats fed with cooked or photoirradiated free VitA, while those fed with cooked or photoirradiated T-VitA-BMC MPs remained at the same absorption level as that of the free VitA(Fig. 4*B*). The encapsulated VitA exposed to cooking and photoirradiation yielded ninefold and 3.5-fold higher absorption than the cooked or photoirradiated free VitA, respectively.

### Absorption of VitA from VitA-BMC-S MPs in Humans.

**Fig. 5.**
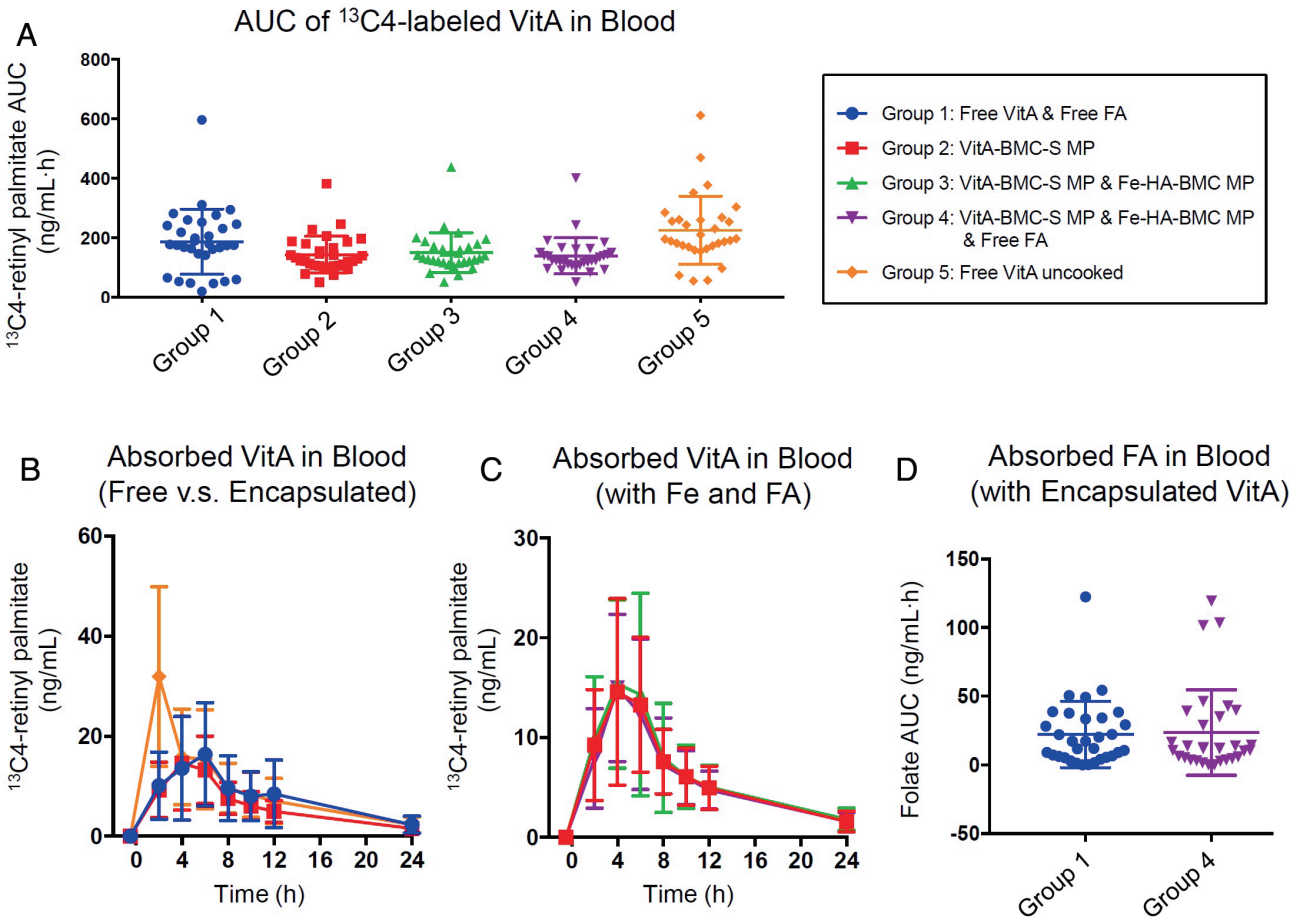
Absorption of VitA and FA in humans from VitA-BMC-S MPs, with or without combination of Fe-encapsulated MPs and free FA in bread (N = 31). VitA absorption was evaluated by measuring plasma ^13^C4-labeled retinyl palmitate (VitA) for 24 h after the test meal and calculating positive incremental AUC. (*A*) AUC values of the ^13^C4-labeled VitA levels in all five test meal groups. Groups 2, 3, and 4 are not significantly different from each other but are significantly different from the reference Groups 1 and 5 (*P* < 0.05). (*B*) *t_max_* and *C_max_* in Groups 1 and 2 are significantly different from Group 5 (*P* < 0.001), which is consistent with the fact that oil promotes the absorption of VitA. (*C*) Absorption curves show no significant difference in *t_max_* or *C_max_* for Groups 2, 3, and 4. (*D*) AUC values of the plasma folate levels in Groups 1 and 4 are not significantly different, demonstrating that BMC does not interfere with free folate absorption. Error bars in this figure represent SD.

To determine the potential to use BMC MPs for VitA food fortification, we designed a randomized controlled cross-over clinical trial to measure blood levels of absorbed ^13^C-VitA from bread fortified with VitA-BMC-S MPs, with or without the coadministration of encapsulated iron sulfate (FeSO_4_) and FA. The entire study contained one screening visit and five test periods with two test visits each at the beginning and end of the test period (*SI Appendix*, Fig. S5). As shown in flowchart (*SI Appendix*, Fig. S6), between May 14, 2018, and August 29, 2018, 67 participants were screened, of which 37 were enrolled and randomly assigned a subject number. During the following five test periods, four subjects withdrew due to scheduling conflicts, and two subjects withdrew due to adverse events which were unlikely to have been related to the study product (details are in the *SI Appendix*). Demographic data for the intent-to-treat (ITT) sample population were obtained from all the 37 randomized subjects, as shown in *SI Appendix*, Table S3. Due to early termination, 32 (for Groups 1–3) and 31 subjects (for Group 4 and 5) contributed to the outcome data, larger than the sample sizes calculated for VitA (N = 23) and FA (N = 30), respectively. There were five meal groups in the study (Table 1)—two control groups of free VitA (Group 1 and Group 5, cook and uncooked, respectively) and three sample groups of encapsulated VitA (VitA-BMC-S MPs in Group 2, VitA-BMC-S MPs + Fe-HA-BMC MPs in Group 3, and VitA-BMC-S MPs + Fe-HA-BMC MPs + free FA in Group 4) (formulations in *SI Appendix*, Table S4). The VitA was added to the dough before baking the bread in Groups 1 through 4, while the free VitA was mixed into the soybean oil spread on the bread in Group 5. The plasma absorption profiles of ^13^C-VitA for the different meal groups are shown in Fig. 5 *B* and *C*. The total absorption levels are determined by calculating the area under the curve (AUC) value for each individual and are displayed in Fig. 5*A*. The averages and 95% CI of the AUC values, the *t_max_* and *C_max_* values, are shown in Table 2.

**Table 1.** Dosing of the fortificants added to the meal Groups 1–5

Group	Study material^*^	VitA dose/ person (mg)	Dose/person of other fortificants (mg)
1	free VitA + free FA (in bread)	1.28	FA: 0.36
2	VitA-BMC-S MP (in bread)		NA
3	VitA-BMC-S MP + Fe-HA-BMC MP (in bread)		Fe: 4^†^
4	VitA-BMC-S MP + Fe-HA-BMC MP + free FA (in bread)		FA: 0·36 Fe: 4
5	free VitA (in dipping oil)		NA

^*^All products were cooked in bread except for Group 5, where free VitA in oil was added to bread after cooking and prior to consumption.

^†^The Fe is encapsulating in Fe-HA-BMC MPs, the preparation of which has been published in our previous study (20). And its formulation is in SI Appendix, Table S4.

**Table 2.** The averaged VitA absorption AUC values, maximal concentration (*C_max_*) of plasma metabolites of retinyl palmitate, and time to maximal concentration (*t_max_*) of plasma metabolites of retinyl palmitate for the five meal groups (N = 31–32)^*^

Group	Study material	AUC (95% mean CI) (ng/mL ⋅ h)	*C_max_* (95% mean CI) (ng/mL)	*t_max_* (95% mean CI) (h)
1	Free VitA + free FA (in bread)	188·66 (149·20, 228·11)^†^	18·82 (15·16, 22·48)^§^	4·94 (4·12, 5·76) ^¶^
2	VitA-BMC-S MP(in bread)	145·54 (123·10, 167·99)^‡^, ^**^	16·61 (13·46, 19·76)^||^	4·00 (3·42, 4·58) ^#^
3	VitA-BMC-S MP + Fe-HA-BMC MP (in bread)	152·10 (128·09, 176·11)	17·99 (14·66, 21·33)	4·19 (3·63, 4·75)
4	VitA-BMC-S MP + Fe-HA-BMC MP + free FA (in bread)	141·22 (118·99, 163·46)	16·34 (13·70, 18·98)	4·06 (3·58, 4·55)
5	Free VitA (in dipping oil)	227·01 (185·07, 268·96)	32·75 (26·16, 39·34)	2·32 (1·94, 2·71)

^*^For Groups 1, 2, and 3, N = 32. For Groups 4 and 5, N = 31. One subject participated part of the study due to early termination.

^†^*P* = 0·0276 (vs. Group 2), ^‡^*P* = 0·0005 (vs. Group 5), ^§^*P* = 0·0209 (vs. Group 2), ^||^*P* < 0·0001 (vs. Group 5), ^¶^*P* = 0·0928 (vs. Group 2; no statistical difference), ^#^*P* < 0·0001 (vs. Group 5) as determined by difference test assessed using *Wilcoxon signed-rank test*.

^**^There was no significant difference in the data between Groups 2 and 4 (*P* > 0·05).

By comparing the *t_max_* and *C_max_* of Groups 1 and 5 (Fig. 5*B*), the free VitA in oil was absorbed faster and to a higher degree than in bread. The VitA absorption from VitA-BMC-S MPs in Group 2 was 145.54 (123.10, 167.99) ng/mL h, and absorption of free VitA in Group 1 was 188.66 (149.20, 228.11) ng/mL h. VitA from VitA-BMC-S MPs was absorbed at 77% compared to free VitA (Group 1). Difference testing shows that encapsulated VitA absorbs at similar levels as the absorption of free VitA (Table 2), indicating that VitA is effectively released from the VitA-BMC-S MPs and that BMC does not significantly hinder absorption. To ensure encapsulated VitA can be used with other BMC MPs for fortification, we tested whether the codelivery of MNs would affect VitA absorption. Groups 2, 3, and 4 show no significant differences in the individual absorption profiles of VitA (Fig. 5 *C* and *D*) and FA (*SI Appendix*, Table S5), when delivered with the different combinations of Fe-HA-BMC MPs, VitA-BMC-S MPs, and FA. This demonstrates that the absorption profiles of VitA and FA are not influenced by interactions with other BMC MPs. Thus, BMC-based encapsulation provides a possible solution for food fortification with multiple MNs, which would otherwise be incompatible with each other or with the food vehicle.

## Discussion

The regions most severely impacted by VAD are sub-Saharan Africa and South Asia, where the prevalence of VAD has risen to 48% and 44%, respectively (6). The high temperature and humidified climate in those areas, as well as the cooking customs (such as boiling soup for long periods of time), may significantly impair VitA food fortification. In our study, we simulated environmental conditions through long-term storage and cooking experiments and found that five factors contribute to the degradation of free VitA: temperature, humidity, light, oxidative reagents, and pH. Encapsulation in VitA-BMC-S MPs increases VitA half-life under all five simulated conditions. For example, in boiling water, VitA-BMC-S MPs preserve about ~62% of VitA, while 90% of the free VitA control degrades within 2 h. VitA degradation is also faster in highly humidified environments. The proton in the acidic aqueous phase catalyzes the degradation of VitA (25). We hypothesize that the tertiary amine in BMC is able to capture the proton (Fig. 1*B*) and keep the pH close to seven. In addition to their outstanding VitA protection, VitA-BMC-S MPs can be produced by scalable processes, which will facilitate their cost-effective implementation. VitA-BMC-S MPs also protect against light exposure. It is possible that the improvement of VitA stability of VitA-BMC-S MPs under photoirradiation is due to the starch outer layer acting as a physical filter. Starch particles are often used in sunscreen for this application (29).

Absorption and biodistribution of VitA from VitA-BMC MPs in a rodent model were comparable to free VitA in the uncooked groups, and absorption was greater in the cooked and photoirradiated groups. These results led to a clinical trial to measure VitA absorption in humans after consuming bread made of VitA-fortified flour with VitA-BMC MPs. In our study, the uncooked VitA mixed with oil (Group 5) had the highest absorption level. However, it is known that oil facilitates the absorption of VitA due to its fat solubility (30). Thus, free VitA baked in bread (Group 1) is the proper absorption reference for experimental groups. Considering that the poor stability of VitA in VitA-fortified oil limits the final bioavailability of VitA after storage and cooking (31), it is important to consider both stability and bioavailability. The results indicate that VitA was released from our VitA-BMC-S MPs and absorbed into the blood at levels that are comparable to free, cooked VitA. Encapsulation using BMC polymer does not significantly slow down VitA absorption as seen by comparing the *t_max_* of Group 1 and Group 2, even though VitA must be released from the MPs. Ideally, we envision the use of BMC MPs for omni-fortification, thereby combating deficiencies of multiple MNs with one MP that can be incorporated into food staples. When other fortificants were added (combinations of encapsulated VitA, encapsulated Fe, and free FA), the presence of BMC did not hinder the absorption of VitA and FA.

To effectively alleviate VAD through food fortification, it is essential to protect VitA from degradation before ingestion and ensure release and subsequent absorption of VitA in the gastrointestinal tract. By using the experimental stability and bioavailability data, we can estimate the overall bioavailability of VitA-fortified bouillon cubes made with VitA-BMC-S MPs and compare against commercial VitA 250 MPs (Table 3). To make an accurate estimate, we account for absorption and assume that VitA-encapsulated MPs undergo a typical use case scenario, including storage at 40°C and 75% rH for 6 mo, and cooking via boiling in water for 3 h. The estimated overall bioavailability of VitA from VitA-BMC-S MPs is about one order of magnitude higher than VitA-250 MPs. This analysis highlights the downstream effects of outstanding stability during storage and cooking, which is a crucial technical feat necessary to increase the regular intake of VitA and combat VAD through fortified food.

**Table 3.** Estimated calculated bioavailability of VitA from food fortified with VitA-BMC-S MPs or commercial VitA 250 MPs

Processes from storage to cooking and to absorption		VitA-BMC-S MPs	VitA 250 MPs
Recovery of VitA in bouillon cubes	After storage at 40°C and 75% rH for 6 mo	75 ± 10%^*^	15 ± 5%^*^
	After cooking in water at 100°C for 3 h	86 ± 3%^*^	41 ± 1%^*^
Relative bioavailability of encapsulated VitA to free VitA		80%^†^	<100%^‡^
Estimated overall bioavailability of VitA		~50%^§^	~6%^§^

^*^Data from stability tests for bouillon cubes fortified with MPs carried out in this work.

^†^Data from absorption study in humans carried out in this work.

^‡^No reported data available. The highest possible value (100%) was assumed.

^§^The calculated bioavailability of VitA = Recovery of VitA after storage × Recovery of VitA after cooking × relative bioavailability from VitA fortified food after ingestion.

We developed a VitA encapsulation technology that produces VitA-encapsulated MPs by a commercially available process. Our VitA MPs can be easily mixed with common food ingredients (such as bouillon cubes and flour). In vitro stability studies highlighted the VitA MPs’ outstanding performance in stabilizing VitA at high temperature and humidity, during storage, cooking, and autoclave treatment—an observation consistent in powder, bouillon, or liquid form. As shown in the rat study, the absorption of VitA from VitA MPs was similar to that of free VitA, and superior to free VitA after cooking or photoirradiation. A clinical trial in women demonstrated rapid release and subsequent absorption of VitA from bread containing VitA MPs, even in the presence of multiple encapsulated MNs. In total, VitA MPs are an effective VitA fortificant that can be produced on a commercial scale, are shelf stable for months, can be used in cooking with other vital MNs, and retain bioavailability after ingestion.

### Materials and Methods

#### Materials.

All materials were purchased from Sigma unless otherwise specified. BMC polymer, poly(butylmethacrylate-co-(2-dimethylaminoethyl)methacrylate-co-methylmethacrylate) (1:2:1), was purchased from Evonik Industries. VitA (VitA-palmitate 1.7 Mio IU/G unstab., Product # 30041032) and Vit 250 MPs (Dry VitA-Palmitate 250 Food Grade, 250,000 IU/g, stabilized with BHT, Product # 30056968) were obtained from BASF. Tritium-labeled VitA (Retinyl [15-3H] palmitate) was produced by American Radiolabeled Chemicals, Inc. SGF was purchased from Ricca Chemical Company.

#### Lab-Scale Preparation of VitA-BMC MPs.

VitA-BMC MPs were prepared by an oil-in-water emulsion method. 10 mg of VitA and 100 mg of BMC were dissolved in 1 mL dichloromethane. The solution was added into 20 mL 1% polyvinyl alcohol (PVA) solution with stirring at a rate of 300 rpm. After stirring at r.t. for 10 min, the obtained emulsion was poured into 100 mL water and stirred at 500 rpm for 10 min to solidify the MPs. The MPs were collected by sedimentation and were thoroughly washed with water three times. The VitA-BMC MPs were dried by lyophilization. 0.1–1 g of VitA-BMC MPs was successfully produced with a production yield of 60%, and the VitA encapsulation efficiency was 73 ± 7%. The production yield is determined by dividing the weight of obtained MPs by the total weight of the feed material (VitA + BMC). The VitA encapsulation efficiency is calculated by dividing the quantified VitA loading by the theoretical VitA loading.

#### Scaled Manufacturing of VitA-BMC-S MPs.

VitA-BMC-S MPs were prepared using a custom spinning disc atomization system at Southwest Research Institute (SwRI) in San Antonio, TX, USA. The reagents, 54 g of BMC and 6 g of retinyl palmitate (VitA), were dissolved into 810 g of degassed dichloromethane. The solution was gravity fed at 50–75 mL/min onto a 4-inch diameter disc spinning at 1,500 rpm and was atomized off the disc 3 ft. above a collection surface coated with an approximately 1/8th inch thick bed of Dry-Flo starch. After 1 h in the starch bed, the starch bed was collected for sieving. A 75-µm sieve was used to remove the excess starch, and a 212-µm sieve was used to remove any oversized agglomerates to yield approximately 40 g of microspheres (yield ~67%).

#### Scaled Manufacturing of VitA-BMC (Aqueous) MPs.

A Niro Mobile Minor pilot-scale spray dryer was used to prepare VitA-BMC (aqueous) MPs in an aqueous system. The feed solution is an emulsion solution containing 210.8 g VitA (stabilized with BHT), 300 g maltodextrin modified starch, 212 g BMC, 10.9 g sodium dodecyl sulfate (SDS), 100 g sulphuric acid (6M), and 1,200g water with pH = 6 at 50°C. This solution was fed at 50°C into the dryer equipped with a nozzle diameter of 1.0 mm. The atomizing pressure at the nozzle was 0.3–0.4 bar. The inlet temperature was 100°C and the outlet temperature was 60°C. The air flow was 80 m^3^/h. After spraying for 38 min, 889 g VitA-BMC (aqueous) MPs was obtained with particle sizes of 20–60 μm.

#### Morphological Characterization of MPs.

The morphology of MPs was characterized by optical microscopy (Olympus MX40) and scanning electron microscopy (JEOL 5910 SEM). The MPs were coated with Pt/Pd before SEM imaging and cut by a single-edge razor blade for the cross-section observation.

#### Size Characterization of MPs.

The size distribution of MPs was measured with a Beckman–Coulter Multisizer III analyzer (Beckman Coulter, Inc.) using ISOTON^®^ II dilute (Beckman Coulter, Inc.) as the diluent and blank. The diameter of the aperture used was 1,000 μm.

#### Determination of VitA Loading in MPs.

The MPs (~10 mg of VitA-BMC and ~20 mg VitA-BMC-S) were dissolved with 1 mL dichloromethane (containing 0.1% BHT). The insoluble starch was centrifuged down at 8,000 × *g* for 5 min. The solution (0.1 mL) was mixed with 0.9 mL acetonitrile. After filtration, the sample solution was analyzed by reverse-phase HPLC. The VitA 250 MPs (~20 mg) was first dissolved in 0.1 mL water to form a white suspension. Then, 0.9 mL THF (with 0.1% BHT) was added. The insoluble additives were centrifuged down at 8,000 × *g* for 5 min. The solution (0.1 mL) was mixed with 0.9 mL acetonitrile. After filtration, the sample solution was analyzed by reverse-phase HPLC. Five replicates for each sample were tested.

#### Reverse-Phase-HPLC Quantification of VitA.

VitA was analyzed by reverse-phase-high-performance liquid chromatography (RP-HPLC) in an Agilent 1,200 HPLC system with a C-18 column (AcclaimTM PolarAdvantage II, 3 μm, 4.6 × 150 mm). The mobile phase was 100% acetonitrile with a flow rate of 0.5 mL/min. The ultraviolet-visible detector was set to a wavelength of 325 nm, and the detection signal of retinyl palmitate was a symmetric single peak at a retention time of 6.23 min. The standard solutions were prepared in 10% dichloromethane (with 0.1% BHT) in acetonitrile. Using peak integration, the linearity of the standard curve holds well in the range of 2.3–300 μg/mL. The calibration curve all produced a linear regression R^2^ value of at least 0.995 or greater. The sample solution for VitA-BMC, VitA-BMC-S, and VitA-BMC (aqueous) MPs was prepared by dissolving the samples in 1 mL dichloromethane (with 0.1% BHT), and then diluting 10-fold with acetonitrile. The sample solution for VitA 250 MPs was prepared by first dissolving the VitA 250 MPs in 0.1 mL water to form an emulsion, then adding 0.9 mL THF (with 0.1% BHT) to extract the VitA, centrifuging down the insoluble ingredients, and at last diluting the supernatant 10-fold with acetonitrile. The standard solutions for VitA 250 MPs were prepared in a 10% mixture solution of 0.1 mL water and 0.9 mL THF (with 0.1% BHT) in acetonitrile. The standard solution and sample solutions were filtered with a syringe filter (polytetrafluoroethylene membrane, pore size 0.2 μm) before injection.

#### Determination of the Antioxidative Activity of VitA-BMC MPs.

The antioxidative activity of VitA-BMC MPs was evaluated using 1,1-diphenyl-2-picryl-hydrazyl (DPPH) assay. VitA-BMC MPs (400 mg) of different loading amount were dissolved in 1 mL ethanol. A DPPH solution (0.1 mL) was mixed with 0.1 mL testing samples (with ethanol as the control). The mixtures were shaken and incubated for 30 min at r.t., and the absorbance of the solution was measured at 517 nm using a microplate reader (Tecan Infinite M1000 Pro). The DPPH radical scavenging activity (%) = [1–(A_sample_/A_control_)] × 100%.

#### Glass Transition Temperature Measurement of VitA-BMC.

The Tg of VitA-BMC mixture was investigated by differential scanning calorimetry (DSC) (DSC8500, Perkin-Elmer, USA). The instrument was calibrated with indium and zinc. VitA-BMC mixtures with loading of VitA (0%, 1.0%, 4.8%, 9.1%, and 16.7%) were prepared and dried under vacuum overnight. The samples (~5 mg) were tested using the following temperature program: holding for 5 min at –20°C, heating from –20 to 90°C, holding for 5 min, cooling from 90 to –20°C, heating from –20 to 90°C, holding for 5 min, and cooling from 90 to –20°C. The heating/cooling rate was 30°C min^–1^. Dry nitrogen was used as purge gas at a rate of 20 mL min^−1^. The second heating cycle was used to determine the Tg, which is the half *C_p_* extrapolated value.

#### In Vitro Release of VitA.

The release profiles of VitA from MPs were studied in three different environments: a) water at room temperature, b) boiling water at 100°C, and c) SGF at 37°C. At predetermined time points, all samples were centrifuged at 4,000 rpm for 5 min, and 900 μl of the supernatant was collected for analysis. Then, the samples were replenished with 900 μl of fresh release medium. The aqueous release medium was lyophilized, and the VitA was extracted with 1 mL dichloromethane. The samples were then prepared for analysis by RP-HPLC.

#### Stability Test in Boiling Water.

MPs or free VitA (all containing ~0.6 g VitA) was dispersed in 1 mL water and then heated at 100°C in a thermomixer at 500 rpm for a predetermined time (0.5, 1, 2, and 5 h). There was a hole in the cap to allow for oxygen exchange. 0.2 mL water was added every 30 min to compensate for evaporation. After centrifuging at 4,000 rpm for 5 min, the supernatant was transferred into a new tube. The supernatant was lyophilized. The VitA in the particles and supernatant was analyzed using RP-HPLC. Because it is dissolved in water, emulsion samples of VitA 250 MPs were lyophilized after boiling in water for a predetermined time, and then the residual VitA was analyzed using RP-HPLC. Three replicates for each sample were tested.

#### Stability Test Under Irradiation.

A 300 W Xenon arc-based solar simulator was purchased from Solar Light (Glenside, PA; airmass (AM) simulator model 16S-300-002). The simulator is equipped with AM0 and AM1.5 filters with a spectral range from 290 to 2,800 nm. The output lens has a working distance of 150 mm and a focus point diameter of 57 mm. The irradiance at the focus point is 670 mW/cm^2^. The irradiance at the focus point is recorded with a PMA 2100 data logging radiometer. A Peltier thermoelectric cooling module with a cooling and heating temperature range from –20 to 100°C (CP-200HT-TT) was used with an external bipolar temperature controller (TC-720-OEM), which was purchased from TE technologies (Traverse city, MI). Solar spectrum at the focus point was measured using a Flame-S-VIS-NIR-ES with a cosine corrector from Ocean Optics (Dunedin, FL).

The MP or free VitA samples (containing ~0.6 g VitA) were placed at the focus point in a borosilicate flat bottom 4-mL scintillation vial on the cold stage in a water bath set at a temperature of 10°C. Samples were irradiated at 670 mW/cm^2^ for 5 min. The irradiance of the sun at sea level on a cloudless day is considered to be 100 mW/cm^2^ (32). Therefore, the samples were irradiated at ~seven times the exposure of sun irradiance at sea level. VitA was extracted from the particles with DCM (0.1% BHT), and the amount of VitA was analyzed by RP-HPLC. Three replicates for each sample were tested.

#### Stability Test Under Autoclave Conditions.

VitA-BMC-S and VitA 250 MPs were dispersed in 2 mL water and placed in an autoclave chamber. Two conditions were tested, 100°C for 15 min and 123°C for 3 min, and each sample had five replicates. Other sterilization parameters were kept constant with a purge time of 2 min, a precharge of 15 psi, a prevacuum vacuum point of 10 lnHg STPNT, a prevacuum vacuum time of 1 min, a half-ramp time of 15 s, a final ramp slope of 9.0 degree/min, the number of prevacuum as two, the number of post vacuum as one, a slow exhaust end point of 0.5 +psi/-lnHg, a slow exhaust ramp of 0.4 psi/min, and a liquid cooling time of 10 min. The whole process ran for approximately an hour. VitA was extracted from MPs with THF (0.1% BHT), and the amount of VitA was analyzed by RP-HPLC.

#### Preparation of Bouillon Cubes with VitA-MPs.

Bouillon cubes (Maggi, Nestle, USA) were ground into fine powder by a mortar and pestle. VitA-encapsulated MPs were weighed and mixed with the bouillon powder to reach a concentration of ~0.655 mg/g VitA in bouillon powder. The bouillon powder was pressed by a tablet presser to reform each bouillon cube. The average weight of the cubes was around 1.8 g, containing ~1.18 mg VitA. The cubes were wrapped with the original Maggie wrapper secured with tape.

#### Stability of VitA-MPs in Bouillon Cubes.

Bouillon cubes were placed in individual 50 mL Falcon tubes and stored at 40°C and 75% RH for long-term stability studies. The storage stability and subsequent cooking stability were measured at 0, 1, and 2 wk and at 1, 2, 4, 6, 8, 10, and 12 mo. To measure VitA storage stability, the bouillon cube was dissolved into 20 mL water, and 4 mL of the resultant solution was used to measure the amount of VitA. The rest of the solution was diluted into 180 mL water and subsequently cooked for 2 h to further test VitA cooking stability after storage. The initial samples (week 0) were cooked for 2, 2.5, 3, 3.5, and 4 h to test the cooking stability over increased cooking time. The initial gross mass of the samples in water was recorded, and additional water was supplemented at each final time point to the preboiled mass. 45 mL was used to measure the amount of VitA. All collected samples were lyophilized and subjected to VitA content analysis. There were five replicates for each point.

#### Extraction and Detection of VitA from Bouillon Cubes.

The VitA was extracted from lyophilized bouillon samples with 3 mL water-tetrahydrofuran (1:9) (containing 0.1% BHT), vortexed, and centrifuged at 2,000 × *g* for 5 min. 0.1 mL supernatant was diluted by acetonitrile (containing 0.1% BHT) to 1 mL. After filtration, the sample was analyzed by RP-HPLC. Five replicates for each sample were tested.

#### Stability Test for Long-Term Storage Conditions.

MPs or free VitA (containing ~ 0.6 g VitA) were placed in 1.5 mL microcentrifuge tubes and stored under four different conditions: a) MPs as dry powders at 40°C and 75% rH, b) MPs as dry powders at 15°C and 75% RH, c) MPs dispersed in DI water at r.t., and d) MPs dispersed in DI water at 4°C. At a predetermined time (1 d, 1, 2, 4, and 8 wk, and 3, 4, and 6 mo), the sampleswere removed and VitA extracted from MPs with DCM (0.1% BHT) for VitA-BMC and VitA-BMC-S MPs and THF (0.1% BHT) for VitA 250 MPs, lyophilized if dispersed in water, and then subjected to RP-HPLC analysis. There were three to five replicates for each data point.

#### Calculation of the Half-Life Time of VitA Degradation.

According to the literature, VitA degradation is assumed to follow first-order reaction kinetics (33, 34). The first-order rate constant (k) was calculated for each sample by plotting the linear regression of ln(1/VitA recovery) versus the time using GraphPad Prism 8.3.0. The degradation rate constant (k) is the slope of the linear fit. The half-life (t_1/2_) (time required for 50% of VitA to degrade) was calculated based on the equation $t_{\frac{1}{2}}=\frac{ln2}{k}$.

#### VitA Absorption Study in Rats.

Animal studies were approved by the Institute Committee for Animal Care and Use (IACUC) and were performed at the Massachusetts Institute of Technology (MIT). Tritium-labeled retinyl palmitate (T-VitA), which is retinyl [15-3H] palmitate (Ci/mmol, 1 mCi/mL in ethanol) (American Radiolabeled Chemicals, Inc.), was used to detect the amount of absorbed VitA in blood and tissue. VitA-BMC MPs were prepared by the O/W emulsion method described above. The loading of T-VitA was quantified by a scintillation counter. See *SI Appendix* for details.

#### Statistical Analysis for Studies In vitro and In Vivo.

All quantitative measurements were performed as at least three independent replicates. The values of the in vitro and in vivo study were expressed as the mean ± SD. Statistical significance was evaluated using the ordinary one-way ANOVA and *t* test with GraphPad Prism 8.3.0 (GraphPad Software, LLC.). A *P* value of <0.05 was considered to be statistically different.

#### Institutional Review Board.

The initial study protocol was approved by IntegReview on September 21, 2017, prior to study commencement and subject recruitment. Committee on the Use of Humans as Experimental Subjects (COUHES) determined that a separate MIT IRB approval was not required. IntegReview is fully accredited by the Association for the Accreditation of Human Research Participation Protection Programs (AAHRPP).

#### Study Design and Participants in Clinic Study.

A randomized, controlled, cross-over human study with one screening visit and five test periods (*SI Appendix*, Fig. S5) was performed in collaboration with and at the facility of Merieux NutriSciences US Biofortis Research (Chicago, IL, USA) to evaluate the absorption of free and encapsulated VitA and free FA in healthy, premenopausal women. This work was approved by IntegReview prior to subject recruitment and study commencement. Informed written consent was obtained from all participants. Details of the design and methods can be found in the *SI Appendix*.

## Supplementary Material

Appendix 01 (PDF)

## Data Availability

All presented data are tabulated and detailed in the main text and the *SI Appendix*. A document containing deidentified individual participant data reported in this article will be available, along with a data dictionary, statistical analysis plan, and analysis tables. Data will be available upon publication for 5 y. Data will be shared for individual-participant-data meta-analysis with other members of the research community who have an affiliation to a recognized medical university. Data will only be shared with investigator support, after approval of a proposal, and with a signed data access agreement. Proposals can be directed to Email address: tangw@scut.edu.cn.

## References

[1] R. Blomhoff, H. K. Blomhoff, Overview of retinoid metabolism and function. J. Neurobiol. **66**, 606–630 (2006). 16688755 10.1002/neu.20242

[2] S. Muthayya , The global hidden hunger indices and maps: An advocacy tool for action. Plos One **8**, e67860 (2013). 23776712 10.1371/journal.pone.0067860 PMC3680387

[3] WHO, Vitamin A Deficiency and its Consequences: A Filed Guide to Detection and Control (WHO,ed. 3, 1995).

[4] P. R. Tepasse , Vitamin A plasma levels in COVID-19 patients: A prospective multicenter study and hypothesis. Nutrients **13**, 11 (2021). 10.3390/nu13072173 PMC8308355 34202697

[5] WHO, Global Prevalence of Vitamin A Deficiency in Populations at Risk 1995–2005 (WHO, 2009).

[6] G. A. Stevens , Trends and mortality effects of vitamin A deficiency in children in 138 low-income and middle-income countries between 1991 and 2013: A pooled analysis of population-based surveys. Lancet Glob. Health **3**, E528–E536 (2015). 26275329 10.1016/S2214-109X(15)00039-X

[7] WHO, Guideline: Vitamin A Supplementation in Infants and Children 6–59 Months of Age (WHO, (2011). 24575452

[8] A. Imdad, E. Mayo-Wilson, K. Herzer, Z. A. Bhutta, Vitamin A supplementation for preventing morbidity and mortality in children from six months to five years of age. Cochrane Database Syst. Rev. **3**, CD008524 (2017). 28282701 10.1002/14651858.CD008524.pub3 PMC6464706

[9] J. Mason, T. Greiner, R. Shrimpton, D. Sanders, J. Yukich, Vitamin A policies need rethinking. Int. J. Epidemiol. **44**, 283–292 (2015). 25306559 10.1093/ije/dyu194

[10] J. B. Mason, D. Sanders, T. Greiner, R. Shrimpton, J. Yukich, Vitamin A deficiency: Policy implications of estimates of trends and mortality in children. Lancet Glob. Health **4**, E21 (2016). 26718802 10.1016/S2214-109X(15)00246-6

[11] J. B. Mason , Effects on vitamin A deficiency in children of periodic high-dose supplements and of fortified oil promotion in a deficient area of the Philippines. Int. J. Vitam. Nutr. Res. **81**, 295–305 (2011). 22419200 10.1024/0300-9831/a000077

[12] N. Failloux, I. Bonnet, E. Perrier, M. H. Baron, Effects of light, oxygen and concentration on vitamin A. J. Raman Spectrosc. **35**, 140–147 (2004).

[13] C. Yan, Utilization of polysaccharide-based high internal phase emulsion for nutraceutical encapsulation: Enhancement of carotenoid loading capacity and stability. J. Functional Foods **84**, 104601 (2021).

[14] M. Rovoli, I. Pappas, S. Lalas, O. Gortzi, G. Kontopidis, In vitro and in vivo assessment of vitamin A encapsulation in a liposome-protein delivery system. J. Liposome Res. **29**, 142–152 (2019). 30187807 10.1080/08982104.2018.1502314

[15] A. Goncalves, B. N. Estevinho, F. Rocha, y Microencapsulation of vitamin A: A review. Trends Food Sci. Technol. **51**, 76–87 (2016).

[16] V. Jenning, S. H. Gohla, Encapsulation of retinoids in solid lipid nanoparticles (SLN (R)). J. Microencapsul. **18**, 149–158 (2001). 11253932 10.1080/02652040010000361

[r17] N. Ghouchi-Eskandar, S. Simovic, C. A. Prestidge, Solid-state nanoparticle coated emulsions for encapsulation and improving the chemical stability of all-trans-retinol. Int. J. Pharm. **423**, 384–391 (2012). 22210001 10.1016/j.ijpharm.2011.12.027

[18] P. A. Murphy, B. Smith, C. Hauck, K. Oconnor, Stablization of vitamin A in a synthetic rice premix. J. Food Sci. **57**, 437–439 (1992).

[19] I. Ezpeleta, Gliadin nanoparticles for the controlled release of all-trans-retinoic acid. Int. J. Pharm. **131**, 191–200 (1996).

[20] A. C. Anselmo , A heat-stable microparticle platform for oral micronutrient delivery. Sci. Transl. Med. **11**, 13 (2019). 10.1126/scitranslmed.aaw3680 31723037

[21] J. Eisele, G. Haynes, K. Kreuzer, C. Hall, Toxicological assessment of anionic methacrylate copolymer: I. Characterization, bioavailability and genotoxicity. Regul. Toxicol. Pharm. **82**, 39–47 (2016). 10.1016/j.yrtph.2016.11.009 27825834

[22] WHO, Assessing the Iron Status of Populations (WHO, 2007).

[23] E. McLean, B. de Benoist, L. H. Allen, Review of the magnitude of folate and vitamin B-12 deficiencies worldwide. Food Nutr. Bull. **29**, S38–S51 (2008). 18709880 10.1177/15648265080292S107

[24] BASF, Dry Vitamin A-Palmitate 250. h​ttp​s:/​/nu​tri​tio​n.b​asf​.co​m/g​lob​al/​en/​hum​an-​nut​rit​ion​/do​wnl​oad​s.​html​#%7​B%2​20%​22%​3A%​5B%​5B%​22p​rod​uct​Id%​22%​2C%​5B%​22%​2F8​799​791​448​206%2F000000000030056968%22%5D%5D%5D%7D. Accessed 28 June 2020.

[25] S. A. Wilkinson, M. D. Earle, A. C. Cleland, Effects of food composition, pH, and copper on the degradation of vitamin A in beef liver puree during heat processing. J. Food Sci. **47**, 844–848 (1982).

[26] D. Moretti, R. F. Hurrell, C. I. Cercamondi, "Chapter 16 - Bouillon Cubes" in Food Fortification in a Globalized World, M. G. V. Mannar, R. F. Hurrell, Eds. (Academic Press, 2018), pp. 159–165.

[27] WHO, WHO Guidelines on Stability Testing of Pharmaceutical Products Containing Well-Established Drug Substances in Conventional Dosage Forms (WHO, 1994). https://apps.who.int/iris/handle/​10665/62169.

[28] R. Blomhoff, M. H. Green, J. B. Green, T. Berg, K. R. Norum, Vitamin A metabolism - new perspectives on absorption, transport, and storage. Physiol. Rev. **71**, 951–990 (1991). 1924551 10.1152/physrev.1991.71.4.951

[29] V. H. P. Infante , Eco-friendly sunscreen formulation based on starches and PEG-75 lanolin increases the antioxidant capacity and the light scattering activity in the visible light. J. Photochem. Photobiol. B Biol. **222**, 112264 (2021). 10.1016/j.jphotobiol.2021.112264 34320457

[30] W. S. White , Modeling the dose effects of soybean oil in salad dressing on carotenoid and fat-soluble vitamin bioavailability in salad vegetables. Am. J. Clin. Nutr. **106**, 1041–1051 (2017). 28814399 10.3945/ajcn.117.153635 PMC5611781

[31] M. Pignitter, Vitamin A is rapidly degraded in retinyl palmitate-fortified soybean oil stored under household conditions. J. Agric. Food. Chem. **62**, 7559–7566 (2014). 25003735 10.1021/jf502109j

[32] H. S. Rauschenbach, “Environments and their effects” in Solar Cell Array Design Handbook: The Principles and Technology of Photovoltaic Energy Conversion (Van Nostrand Reinhold, New York, ed.**1**, 1980), p. 409.

[33] Y. O. Li, J. Lam, L. L. Diosady, S. Jankowski, Antioxidant system for the preservation of vitamin A in Ultra Rice. Food Nutr. Bull. **30**, 82–89 (2009). 19445263 10.1177/156482650903000109

[34] K. Pentieva, The short-term bioavailabilities of [6S]-5-methyltetrahydrofolate and folic acid are equivalent in men. J. Nutr. **134**, 580–585 (2004). 14988450 10.1093/jn/134.3.580

