# Supplementary material for: Enhanced stability and clinical absorption of a form of encapsulated vitamin A for food fortification
Source: Proc Natl Acad Sci U S A. 2022 Dec 12;119(51):e2211534119. doi: 10.1073/pnas.2211534119 (PMC9907063; doi:10.1073/pnas.2211534119)
Supplement: Supplementary file 1 — Appendix 01 (PDF) [file pnas.2211534119.sapp.pdf]

Supporting Information for

Enhanced stability and clinical absorption of a new form of encapsulated vitamin A for food fortification

Wen Tang,[1,2] Jia Zhuang,[1] Aaron C. Anselmo,[1,3] Xian Xu,[1] Aranda Duan,[1] Ruojie Zhang,[1] James L. Sugarman,[1] Yingying Zeng,[1] Evan Rosenberg,[1] Tyler Graf,[1] Kevin J. McHugh,[1,4] Stephany Y. Tzeng,[1,5] Adam M. Behrens,[1] Lisa E. Freed,[1] Lihong Jing,[1, 7] Surangi Jayawardena,[1] Shelley B. Weinstock,[6] Xiao Le,[1] Christopher Sears,[8] James Oxley,[9] John L. Daristotle,[1] Joe Collins,[1] Robert Langer,[1*] and Ana Jaklenec[1*]

1. David H. Koch Institute for Integrative Cancer Research. Massachusetts Institute of Technology, Cambridge, MA, 02139, USA.
2. South China Advanced Institute for Soft Matter Science and Technology, School of Emergent Soft Matter, South China University of Technology, Guangzhou 510640, China.
3. Division of Pharmacoengineering and Molecular Pharmaceutics, Eshelman School of Pharmacy, University of North Carolina at Chapel Hill, Chapel Hill, NC 27599, USA
4. Rice University, Houston, TX, 77005, USA
5. Department of Biomedical Engineering, Johns Hopkins University, Baltimore, MD 21231, USA
6. Weinstock Nutrition, LLC, South Orange, NJ, 07079, USA
7. Key Laboratory of Colloid, Interface and Chemical Thermodynamics, Institute of Chemistry, Chinese Academy of Sciences, Bei Yi Jie 2, Zhong Guan Cun, Beijing 100190, China.
8. Independent Scholar, Belmont, Massachusetts, 02478, USA
9. Southwest Research Institute, San Antonio, TX, 78238, USA

Ana Jaklenec and Robert Langer
Email: jaklenec@mit.edu and rlanger@mit.edu

This PDF file includes:

Supporting text
Figures S1 to S6
Tables S1 to S5
SI References

Supporting Information Text

Methods for VitA absorption study in rats

Female Wistar rats (~250g) were purchased from Charles River Laboratories. There was neither diet-control nor fasting before the experiment. The rats were divided into six groups of n=6. The six groups were fed with either (a) Free T-VitA, (b) T-VitA-BMC, (c) Free T-VitA-cooked, (d) T-VitA-BMC-cooked, (e) Free T-VitA-light, or (f) T-VitA-BMC-light. The samples in groups c and d were heated in boiling water at 100 ^{o}C for 1 hour and then lyophilized. Samples in groups e and f were irradiated by sunbeam (7x sun intensity) for 5 min on a sample stage at 10 ^{o}C. The ethanol solution of Free T-VitA was added into water and vortexed to form a well-dispersed emulsion. The T-VitA-BMC was dispersed in water and vortexed to form a suspension. Each rat was fed with 10 μCi T-VitA in its free form or encapsulated MPs dispersed in 350 μL water and 15 μL ethanol.

The oral administration was delivered by gavage to the restrained rats using a ball-tipped stainless-steel gavage needle. The residual T-VitA in the syringe and gavage needle was quantified by scintillation counter to calculate the actual feeding amount of T-VitA for each rat. After 24 hours, the rats were euthanized by CO_2, and their stomach, small intestine, liver, lung, spleen, kidney, heart and eye tissues were collected for analysis.

T-VitA radioactivity measurement. The radioactivity in the samples was quantified by liquid scintillation counting with a TriCarb liquid scintillation analyzer (with a counting efficiency of 36% for ^{3}H in Hionic-Fluor liquid scintillation cocktail and 65% for ^{3}H in Ultima Gold™ F liquid scintillation cocktail). To determine the loading of T-VitA in MPs, the T-VitA-BMC was first dissolved in 1 mL dichloromethane, and then 5 μL of the solution was mixed with 10 mL Ultima Gold™ F liquid scintillation cocktail (PerkinElmer Inc.). Standard solutions were prepared by mixing the 1 mCi/mL T-VitA solution in ethanol with the same amount of liquid scintillation cocktail. A blank was prepared by mixing dichloromethane with the Ultima Gold™ F liquid scintillation cocktail. The solution was allowed to set for at least 1 hour before reading. The concentration of radioactivity in the sample solution, c_s, was calculated by the equation

$$c_s = \mathrm{c}_0 \times \frac{R_S - R_B}{R_0 - R_B} \qquad \text{(Equation 1)}$$

where c_0 is the concentration of standard solution in μCi/mL, R_0 is the counts per minute for the standard solution, R_B is the counts per minute for the blank, and R_s is the counts per minute for the sample solution.

For quantification of T-VitA, the blood and tissue samples were dissolved with SOLVABLE™ (PerkinElmer Inc.) following a standard protocol. 1 mL T-VitA solution in SOLVABLE™ was mixed with 10 mL Hionic-Fluor liquid scintillation cocktail as sample solution. 200 μL blood from a rat that was not fed T-VitA was prepared in the same manner and served as the blank solution. A standard solution was prepared from a known amount of T-VitA in 200 μL non-radioactive blood. The concentration was calculated using Equation 1. The total blood volume for each rat was calculated based on the total weight of each rat. The percentage of T-VitA in blood to the total feeding is calculated by equation

$$\mathrm{T-VitA\ in\ Blood\ (\%)} = \frac{c_S \times V_s}{R_F} \qquad \text{(Equation 2)}$$

where c_s is the concentration of radioactivity in the blood in μCi/mL, V_s is the total blood volume of the rat in mL, and R_F is the actual amount of radioactivity fed to the rat. Similarly, whole tissues were weighed, and the percentage of T-VitA in the tissues to the total feeding is calculated. The absorbed T-VitA is the sum of the T-VitA in liver, small intestine, lung, kidney, heart, spleen and eye.

Methods for human clinical trial

Subject inclusion criteria. Subject inclusion criteria were as follows: 18-30 years of age, healthy, premenopausal females, a BMI between 18.5-29.9 kg/m^2, no dietary supplement intake, and maintains a regular diet and physical activity pattern while abstaining from restricted foods and vigorous exercise. Exclusion criteria specifies things in opposition to the inclusion criteria as well as recent history of anemia, recent blood transfusions or donations, a plasma homocysteine concentration >12 µmol/L, or a presence/history of cardiac, renal, hepatic, endocrine, cardiovascular, pulmonary, pancreatic, neurologic, immunologic, dermatologic, rheumatic, gastrointestinal, active infection, cancerous, and/or biliary conditions. Further exclusions include VitA deficiency, folate deficiency, recent trauma/surgical events, uncontrolled hypertension, chronic use of anti-inflammatory drugs/antacids/medicines that interfere with VitA/FA/Fe absorption, use of hormone therapy, pregnancy, an allergy/sensitivity to any of the food ingredients used in the study meals, or alcohol abuse.

Randomization and masking in clinic study. A randomization sequence prepared by a statistician was uploaded onto Medrio eCRF platform (Medrio Inc, San Francisco, CA). When a subject was determined to be eligible for the study, a randomization number was assigned to the subject through randomization module of the Medrio platform. The randomization number was recorded in the subject's source documentation.

Sample size in clinic study. The evaluable sample sizes calculated were N = 30 for folic acid and N = 23 for vitamin A. To account for attrition, a total of 37 subjects were randomized.

Sample size calculation in clinic study. For vitamin A metabolites, sample size calculation was performed in consultation with Dr. Georg Lietz (Newcastle University, UK) using the following parameters: i) Reference group: Mean AUC = 0.21, SD = 0.09; ii) Estimated values for comparator group: Mean AUC = 0.147, SD = 0.09, assuming 30% difference with reference group and SD kept constant; iii) Alpha = 0.05 and iv) Power = 90%. The resulting evaluable sample size is 23 subjects. For folate, sample size calculation was done assuming similar mean and SD AUC from previous study (*34*) with the following parameters: i) Reference group: Mean AUC = 0.21, SD = 0.09; ii) Estimated values for comparator group: Mean AUC = 146 nmol/L×h, SD = 58 nmol/L×h, assuming 20% difference with reference group and difference between groups is 10 nmol/L×h; iii) Alpha = 0.05 and iv) Power = 80%. The resulting evaluable sample size is 30 subjects.

Procedures in clinic study. Each subject consumed one meal within 10 min per visit, which consisted of a fortified bread bun served with 10 g (± 2.5%) of a soybean oil spread and 240 mL of water. As shown in Figure S5, each subject completed a total of 5 visits to consume one of each of the 5 test meals in a randomized, blinded order. The meals, the fortificants used in the meals, and the dose levels are listed in Table 1. In all meal groups, 1.28 mg (the recommended daily allowance for VitA for pregnant women) of isotopically-labeled VitA (vitamin A palmitate (12,13,14,20-^{13}C4) stabilized by 0.5% butylated hydroxytoluene (BHT); produced at Buchem B.V., The Netherlands) was used to fortify the meal. The free VitA in Group 5 was added to the oil spread and therefore was not baked; it is the uncooked control. Group 1 is the unencapsulated free VitA that was baked into the bread with free FA; it is the cooked control with stabilizer BHT. The VitA in Group 2 to 4 is encapsulated in VitA-BMC-S MPs with or without codelivery of encapsulated Fe (formulation in Table S4) and unencapsulated free folic acid. Following a typical Senegalese bread recipe, each bun consisted of 100 g of bread dough (wheat flour, salt, dry yeast, water, and fortificants) that was allowed to rise for 45 min at 30 ^{o}C and 80% relative humidity. The buns were then baked at 190 ^{o}C for 20 min. The fortificants were specifically kneaded into the center portion of the bread dough buns to reduce VitA loss.

A 48-h Diet Record was dispensed to confirm compliance with adherence to a low-folate/folic acid diet throughout the 48-h period prior to test visits. A half hour prior to the study meal consumption, the subject gave blood for baseline measurements of retinyl palmitate and plasma

folate. After meal consumption, the subject gave blood at designated timepoints in order to fulfill the needs for plasma folate analyses at t = 1, 1.5, 2, 3, 4, 6, and 8 h ± 5 min and for plasma retinyl palmitate analyses at t = 2, 4, 6, 8, 10, and 12 h ± 5 min. No food was ingested after study meal consumption for the subsequent 4 h period, but *ad libitum* water consumption was allowed for the remainder of the visit following the 2 h blood draw. Subjects were administered a standard low-folate/folic acid, low-vitamin A lunch (immediately following the t = 4 h blood draw), a standard low-folate/folic acid, low-vitamin A snack (immediately following the t = 6 h blood draw) and a standard low-folate/folic acid, low-vitamin A dinner (at t = 10 h). Subjects were instructed to consume the meal/snacks within 30 min and to eat until comfortably full. The meals and snack (choices and amounts) were replicated at subsequent visits. A washout window of 5-7 days followed each test meal visit, and each subject was designated to complete all 5 meal groups.

Outcomes in clinic study. The retinyl palmitate was extracted from the plasma samples using hexanes and quantified by LC-MS-MS. The folate levels in the plasma samples were determined using the commercially available microbiological kit (ALPCO, ref# KIF005) in a 96-well format. Samples were analyzed by Eurofins Craft Technologies. Outcome variables included the following: i) positive incremental area under the curve (AUC) for plasma folate from 0 to 8 h, ii) AUC for plasma ^{13}C4-retinyl palmitate from 0 to 24 h, iii) maximal concentration (C_{max}) of plasma ^{13}C4-retinyl palmitate and plasma folate, and iv) time to maximal concentration (t_{max}) of plasma retinyl palmitate and folate. For plasma folate, the bioequivalence and difference tests were performed by comparing: Group 1 vs. Group 5, Group 2 vs. Group 5, Group 2 vs. Group 4, Group 1 vs. Group 4, and Group 1 vs. Group 2. For plasma retinyl palmitate, the bioequivalence and difference tests were performed by comparing: Group 1 vs. Group 5, Group 2 vs. Group 5, Group 3 vs. Group 5, Group 4 vs. Group 5, Group 2 vs. Group 4, and Group 1 vs. Group 2. For all the aforementioned comparisons, only difference test was performed for t_{max}.

Statistical analysis in clinic study. All statistical analyses were conducted using SAS for Windows (version 9.2, or higher, Cary, NC) and/or R 3.3.1 (R Core Team 2016). Thirty-one subjects completed all 5 test meals of the study (in addition to the 31, 1 subject was lost to an adverse event that was medically deemed unrelated to the study, 1 subject was lost to withdrawal of consent, and 4 subjects were lost because they failed to follow-up). Intent-to-treat population data with outliers was used for all analyses (n=31). The difference between a pair of meal groups was assessed using *Wilcoxon signed-rank test* when the normality assumption was not satisfied. When the normality assumption was satisfied, difference was assessed using unpaired *t-test* (for equal variance) and *Welch's t-test* (for unequal variance).

Role of funding source in clinic study. The funders had no role in study design, data collection, data analysis, data interpretation, or writing of the report. The corresponding author had full access to all the data in the study and had final responsibility for the decision to submit for publication.

Adverse events and other clinical observations in clinic study. Adverse event (AE) inquiries occurred at the beginning (Visits 2 through 11; days 0 through 29) and end (Visits 2, 4, 6, 8, and 10; days 0, 7, 14, 21, and 28) of the study visits. Subjects were asked an open-ended question (e.g. "since you were last asked, have you noticed any changes in the way you feel?") for each AE assessment.

A total of 28 clinical observations were reported by 14 subjects during the study. Six of these (nausea, vomiting, bloating, diarrhea, flatulence) were judged as possibly related to the study product and the others were judged as not related to study product by the Clinical Investigator. All adverse events were judged to be mild to moderate, except for two (nausea and vomiting) reported by Subject 002 which were judged to be serious by the Clinical Investigator. Subject 002 experienced nausea and vomiting during Visit 2 and did not complete the visit. During a follow up call, the subject reported being admitted to the hospital and provided IV fluids and treated for nausea/vomiting. Additional subsequent attempts to obtain follow-up information failed due to subject being non-responsive and subject was considered lost to follow-up.

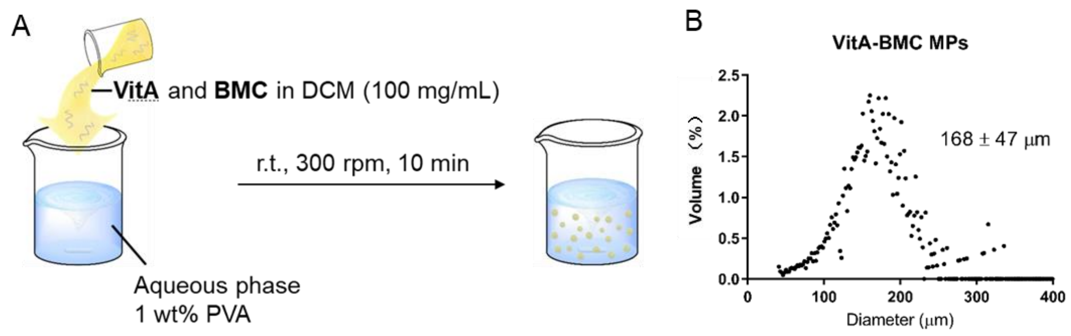

Fig. S1. The lab-scale preparation of VitA-BMC MPs and their size distribution. (A) VitA-BMC MPs were prepared by oil-in-water emulsion method. (B) The size distribution of VitA-BMC MPs was characterized by Coulter counter.

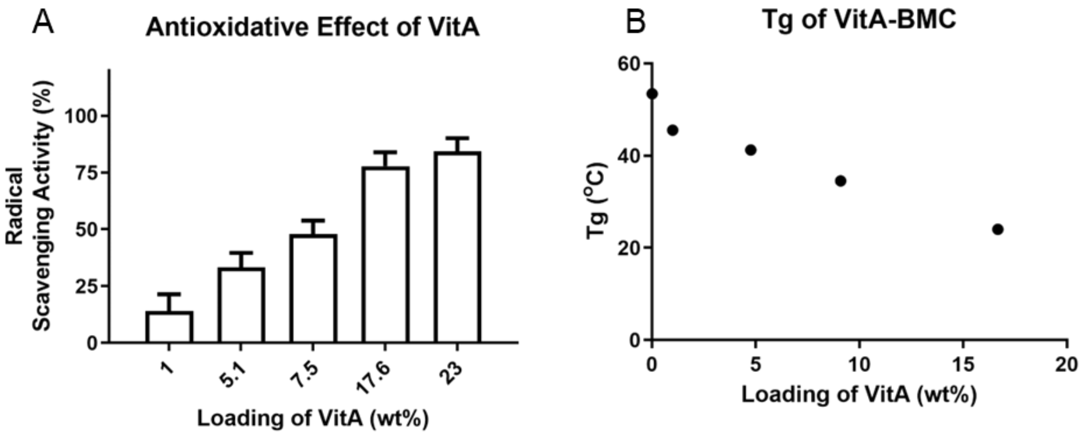

Fig. S2. VitA shows a concentration-dependent antioxidative effect and decreases the Tg of VitA-BMC MPs. (A) The anti-oxidative activity of VitA-BMC MPs of different loading amount was using 1,1-diphenyl-2-picryl-hydrazyl (DPPH) assay. (B) The glass transition temperature (Tg) of VitA-BMC MPs of different loading amount was measured by differential scanning calorimetry (DSC).

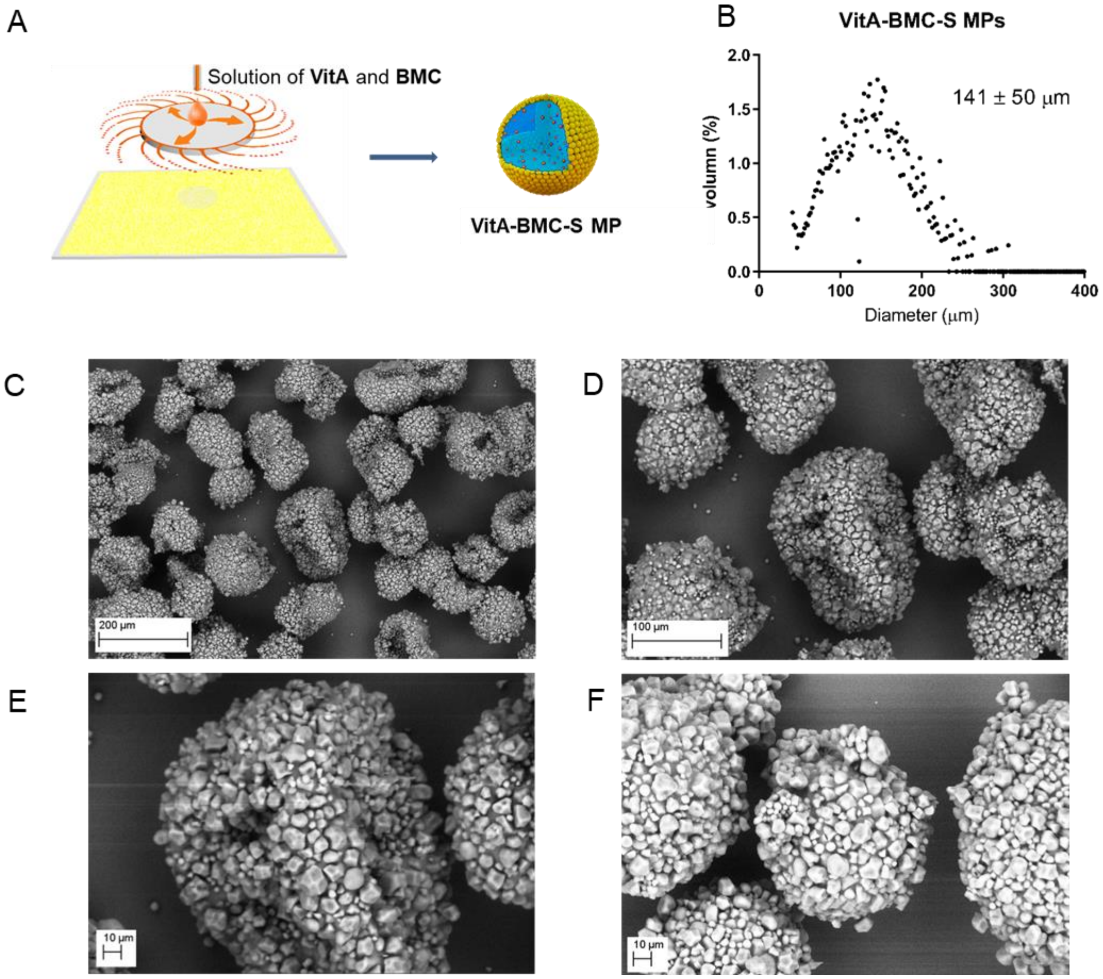

Fig. S3. The pre-manufacture scale preparation of VitA-BMC-S MPs, their size distribution and SEM images. (A) VitA-BMC MPs were prepared by spinning disk. (B) The size distribution of VitA-BMC-S MPs was characterized by Coulter counter. (C-F) SEM images of VitA-BMC-S MPs (scale bars as labeled).

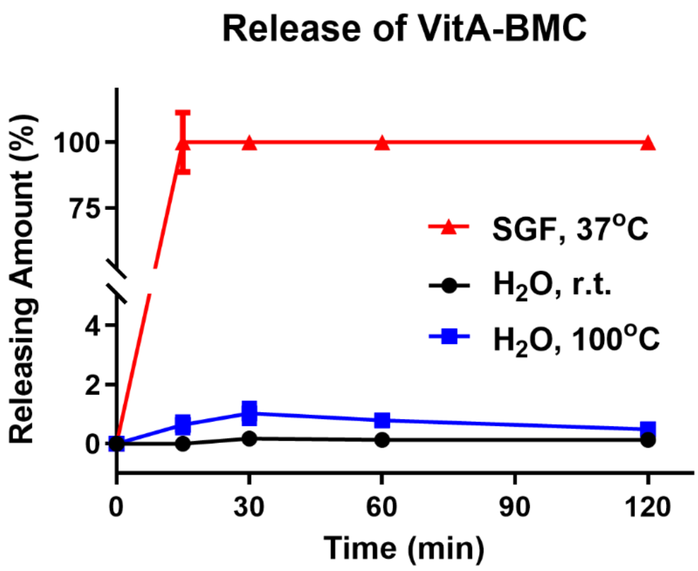

Fig. S4. The release of VitA-BMC MPs in simulated gastric fluid (SGF) at 37 °C, and H_2O at room temperature (r.t.) and 100 °C, respectively.

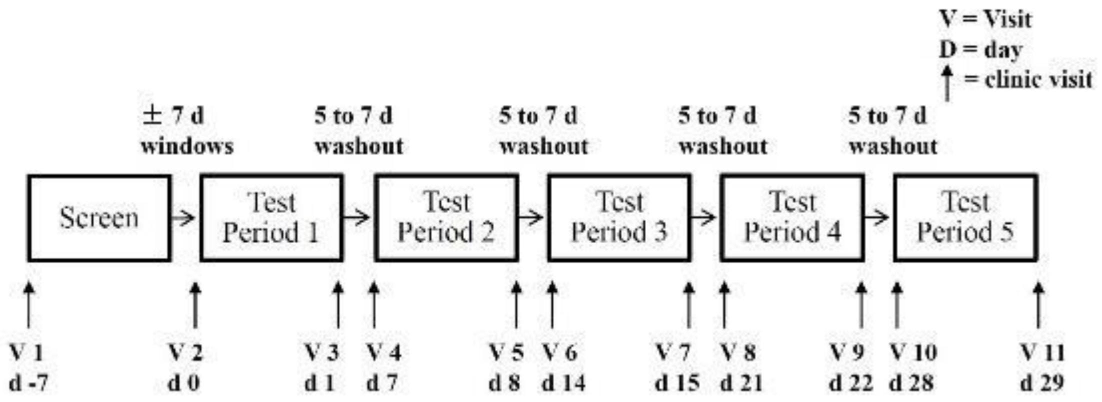

Fig. S5. A randomized controlled crossover study was designed to assess the VitA bioavailability of a fortified bread meal. The study includes one screening visit (Visit 1; day -7) and five test periods [Test Period 1 (Visits 2 and 3; days 0 and 1); Test Period 2 (Visits 4 and 5; days 7 and 8); Test Period 3 (Visits 6 and 7; days 14 and 15); Test Period 4 (Visits 8 and 9; days 21 and 22); and Test Period 5 (Visits 10 and 11; days 28 and 29)].

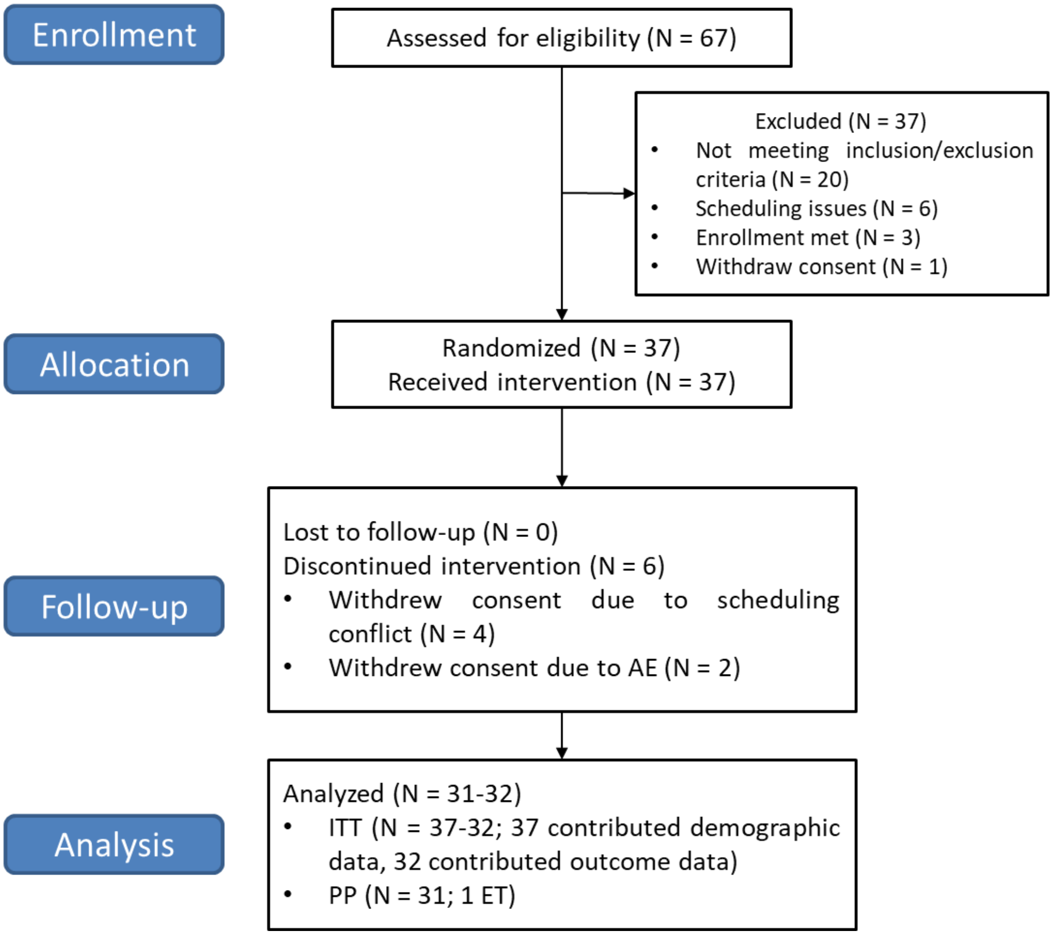

Fig. S6. Trial profile. Subjects were healthy premenopausal women between 18 and 30 years of age. Abbreviation: AE, adverse event; ET, early termination; ITT, intent-to-treat; PP, per protocol; N, sample size.

Table S1. The formulation, loading, encapsulation efficiency and stability of VitA in various formulations of lab-scale produced VitA-BMC MPs.

Formulation		VitA loading in particles (µg/mg)	VitA loading efficiency (%)	Recovery after 100 ºC for 2 h (%)
VitA (mg)	BMC (mg)			
1·3	100	10 ± 1	77 ± 8	56 ± 10
5·0	100	44 ± 4	88 ± 7	66 ± 2
10·0	100	73 ± 7	73 ± 7	80 ± 3
20·0	100	175 ± 1	87.5 ± 0.5	61 ± 1
25·0	100	229 ± 26	91 ± 10	50 ± 13

Table S2. Formulation of MPs used in cooking and long-term storage stability studies.

**VitA-encapsulated MPs**	**Loading of VitA in MPs**		**Other ingredients in the MPs**
	**(wt%)**[a]	**IU/g** [b]	
VitA-BMC	7·5 ± 0·4	~136,000	~90 wt% BMC
VitA-BMC-S	3·7 ± 0·1	~67,000	34 wt% BMC, 62% Starch
VitA-BMC (aqueous)	16·5 ± 0·3	~ 300,000	BMC+SDS (~20%), Vitamin A+BHT (~20%), sugar source (~20%), powdering + residual water (~30%)
VitA 250	17·3 ± 0·6	~ 314,000	BHT, starch, sucrose, medium chain triglyceride, silicon dioxide, sodium ascorbate, sorbic acid, sodium benzoate[6]

[a] The weight of VitA is in the form of retinal palmitate. The degraded VitA was not included.

[b] 1 IU = 0.3 μg of retinol = 0.55 μg of retinal palmitate.

Table S3. Baseline characteristics of enrolled subjects in the intention-to-treat (ITT) population [a].

**Characteristics**		**All randomized subjects ITT population (N = 37)**	**Randomized subjects who contributed outcome data (N = 32[b])**
Age (years)		22·73 (0·67)	22·22 (0·71)
Race	White	28 (75·7 %)	25 (78·1 %)
	Black or African American	4 (10·8 %)	3 (9·4 %)
	American Indian or Alaskan Native	1 (2·7 %)	0 (0·0 %)
	Asian	3 (8·1 %)	3 (9·4 %)
	Native Hawaiian or Other Pacific Islander	0 (0·0 %)	0 (0·0 %)
	Multi-racial	1 (2·7 %)	1 (3·1 %)
Ethnicity	Hispanic or Latino	12 (32·4 %)	11 (34·4 %)
	Not Hispanic or Latino	25 (67·6 %)	21 (65·6 %)
BMI (kg/m^2)		23·51 (0·47)	23·57 (0·51)
Plasma Homocysteine (μmol/L)		10·16 (2·73)	7·46 (0·33)
MTHFR genotype [c]	Negative, Negative	8 (21·6 %)	6 (18·8 %)
	Negative, Heterozygous	6 (16·2 %)	5 (15·6 %)
	Negative, Homozygous	6 (16·2 %)	9 (28·1 %)
	Heterozygous, Negative	9 (24·3 %)	9 (28·1 %)
	Heterozygous, Heterozygous	5 (13·5 %)	5 (15·6 %)
	Homozygous, Negative	2 (5·4 %)	2 (6·3 %)
	Not available	1 (2·7 %)	0 (0·0 %)

[a] Data are Mean (SEM) or N (%). SEM is standard error of the mean.
[b] This is a subset of the ITT population which consists only of subjects who contributed outcome data, whether or not they completed the study in its entirety.
[c] MTHFR mutation C. 1286A → C, MTHFR mutation C.665C→ The N of subjects in the following T. MTHFR genotypes are all 0: Heterozygous, Homozygous; Homozygous, Heterozygous; and Homozygous, Homozygous.

Table S4. Process design formulation parameters and loadings for MPs used in the human study.

<table>
<tr><th>MPs</th><th colspan="4">Process Design</th><th>Loading Amount of MN</th></tr>
<tr><td rowspan="3">VitA-BMC-S MPs</td><td colspan="4">Feed of materials in Spinning Disc</td><td rowspan="3">34 ± 2·4 VitA mg/g MPs</td></tr>
<tr><td colspan="2">VitA (g)</td><td colspan="2">BMC (g)</td></tr>
<tr><td colspan="2">54</td><td colspan="2">1026</td></tr>
<tr><td rowspan="3">Fe-HA-BMC MPs[a]</td><td colspan="2">Feed of materials in Spray Dry</td><td colspan="2">Feed of materials in Spinning Disc</td><td rowspan="3">31·9 ± 0·7 Fe mg/g MPs</td></tr>
<tr><td>$FeSO_4$ Feed (g)</td><td>HA Feed (g)</td><td>HA-Fe Feed (g)</td><td>BMC Feed (g)</td></tr>
<tr><td>3·78</td><td>1·89</td><td>3·57</td><td>0·32</td></tr>
</table>

[a] HA stands for hyaluronic acid. The result of iron absorption has been published.[20]

Table S5. Folic acid absorption with or without encapsulated VitA and encapsulated iron (n=31). [a]

Group	Group Name	AUC (95% Mean CI) (ng/mL·h)	C_{max} (95% Mean CI) (ng/mL)	t_{max} (95% Mean CI) (h)
1	Free VitA + Free FA (in bread)	23·44 (14·26, 32·61) [a]	37·09 (30·03, 44·15)	3·26 (2·64, 3·87)
4	VitA-BMC-S MP + Fe-HA-BMC MP + Free FA (in bread)	24·42[b] (12·71, 36·14) [a]	39·42 (32·21, 46·64)	2·87 (2·41, 3·33)

[a] No significant differences observed between Group 1 and Group 4.
[b] n = 30. Observations excluded when positive incremental AUC is 0.

SI References

20. A. C. Anselmo, X. Xu, S. Buerkli, Y. Y. Zeng, W. Tang, K. J. McHugh, A. M. Behrens, E. Rosenberg, A. R. Duan, J. L. Sugarman, J. Zhuang, J. Collins, X. G. Lu, T. Graf, S. Y. Tzeng, S. Rose, S. Acolatse, T. D. N. Section, X. Le, A. S. Guerra, L. E. Freed, S. B. Weinstock, C. B. Sears, B. Nikolic, L. Wood, P. A. Welkhoff, J. D. Oxley, D. Moretti, M. B. Zimmermann, R. Langer, A. Jaklenec, A heat-stable microparticle platform for oral micronutrient delivery. *Sci. Transl. Med.* **11**, 13 (2019).
